# Editorial: Systemic resistance and defense priming against pathogens

**DOI:** 10.3389/fpls.2023.1267513

**Published:** 2023-08-22

**Authors:** Ho Won Jung, Nicolás M. Cecchini

**Affiliations:** ^1^ Department of Applied Bioscience, Dong-A University, Busan, Republic of Korea; ^2^ Department of Molecular Genetics, Dong-A University, Busan, Republic of Korea; ^3^ Departamento de Química Biológica Ranwel Caputto, Centro de Investigaciones en Química Biológica de Córdoba (CIQUIBIC), CONICET, Universidad Nacional de Córdoba, Córdoba, Argentina

**Keywords:** systemic resistance, defense priming, long-distance mobile signals, immunological memory, pathogens, immunity

Plant diseases pose significant challenges to global agriculture, threatening food security and economic stability. In response to pathogen attacks, plants have evolved a notable immune program(s) known as systemic resistance and the associated defense priming event (Ryals et al., 1996; Conrath, 2011; De Kesel et al., 2021). This phenomenon involves the activation of long-lasting and broad-spectrum disease resistance, which is usually characterized by the plant’s ability to exhibit “immunological memory” and mount a rapid and efficient response upon recurring infections (Martinez-Medina et al., 2016; Sharrock and Sun, 2020). Thus, manipulation of the acquired resistance is a worthy strategy for protecting plants from pathogen infection. To aim it, understanding the mechanisms and signals underlying systemic resistance and defense priming event is crucial for developing sustainable and high-yielding agricultural practices.

The establishment and maintenance of defense priming involve various processes, including epigenetic alterations, accumulation of inactive signaling factors, and changes in the levels of immune receptors, *e.g.*, pattern-recognition receptors (Beckers et al., 2009; Tateda et al., 2014; Cecchini et al., 2015; Conrath et al., 2015; Tsuda and Somssich, 2015; Baum et al., 2019; Jiang et al., 2021; Sheikh et al., 2023). Different systemic resistance programs are activated depending on the specific stimulus in different plant organs/tissues and the long-distance mobile signals implicated (De Kesel et al., 2021). While several systemic signals have been proposed and identified in model and crop plants, our current knowledge about this phenomenon remains predominantly descriptive, with only a few mechanistic insights. To address this knowledge gap, the collection of articles published in the Research Topic on ‘*Systemic Resistance and Defense Priming Against Pathogens*’ deepens our understanding of the signaling pathways, genetic and epigenetic mechanisms, defense signals, exogenous inducers, and factors associated with systemic resistance and defense priming (**Figure 1**).

**Figure 1:**
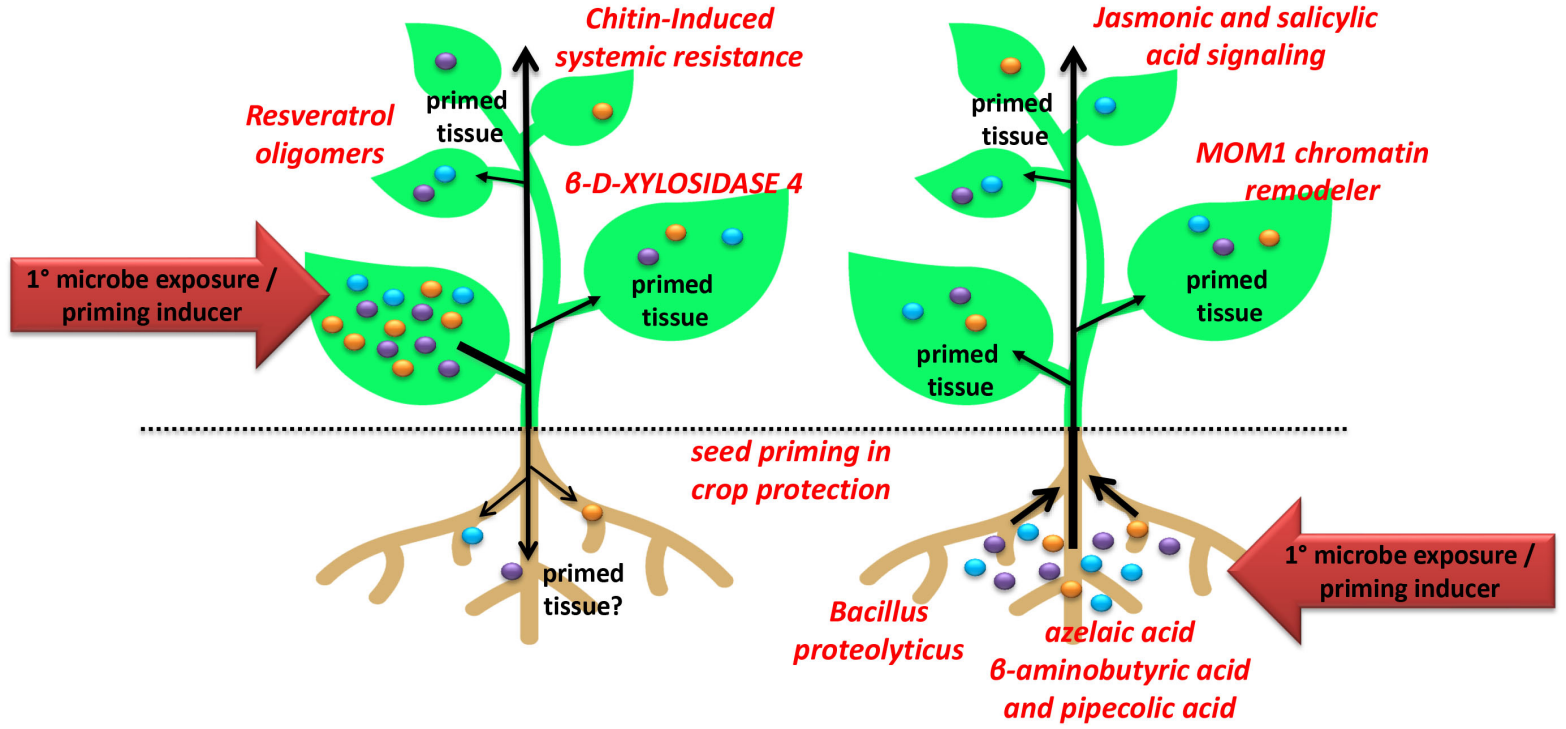
Illustration showing the contributions to the Research Topic on ‘Systemic Resistance and Defense Priming Against Pathogens’.

The study by Ojeda-Rivera et al. investigated the molecular mechanisms underlying resistance to the devastating cotton pathogen, the Root-Knot Nematode. Through genome-wide comparative analysis, the researchers identified a constitutive state of defense transcriptional behavior in the roots of resistant cultivars that shows characteristics of a primed state. This study also sheds light on the role of jasmonic and salicylic acid signaling in nematode resistance and provides potential candidate genes for the development of resistant cotton varieties through defense priming establishment.

The potential of resveratrol oligomers, plant-produced natural compounds, as anti-virulence and immune-priming agents was reported (Kang et al.). These compounds can inhibit bacterial motility and attenuate the formation of the bacterial type III secretion system. Furthermore, resveratrol oligomers induce enhanced local immune responses to virulent *Pseudomonas* infection. This research highlights the promising role of plant-produced natural products, including phytoalexins, as priming inducers beyond antimicrobial properties for disease management. On the other hand, Takagi et al. investigated the systemic induction of disease resistance in rice against *Bipolaris oryzae* through non-plant-derived chitin supplementation. The study revealed alterations in cell-wall biogenesis and the involvement of lysin motif-containing chitin receptors of plants in the expression of chitin-induced systemic response. These findings also provide valuable insights into the mechanisms underlying chitin-induced systemic disease resistance in rice. Similarly, another work shows how *Bacillus proteolyticus* OSUB18 strain triggered systemic resistance enhancing resistance against *P. syringae* and *Botrytis cinerea* in *Arabidopsis* plants when root-drench applied (Yang et al.). These findings provide the potential of the OSUB18 strain as a systemic resistance inducer for effective disease management. These studies strongly support the use of plant- and microbe-derived compounds, along with long-distance mobile signals for systemic resistance to trigger systemic resistance and defense priming.

Other studies explore the role of specific proteins, such as β-D-XYLOSIDASE 4 (BXL4) in systemic immune signaling and the *Arabidopsis* chromatin regulator “Morpheus Molecule 1” (MOM1) in defense priming induction. The study by Bauer et al. explored the role of BXL4 in systemic immune signaling in *Arabidopsis* and demonstrated that this enzyme plays a central role in the well-known systemic acquired resistance program (Ryals et al., 1996). This study also reveals the involvement of cell wall metabolic changes uncovering the complexity of systemic immunity regulation. The study by Miranda de la Torre et al. demonstrated that MOM1 negatively regulates the defense priming induced by the important primed state inducers azelaic acid, β-aminobutyric acid, and pipecolic acid (Zimmerli et al., 2000; Jung et al., 2009; Návarová et al., 2012). This study highlights the importance of the priming of epigenetic modifications regulating immune receptor gene expression as a form of immunological memory. Together, these investigations provide valuable mechanistic insights into the complex networks governing systemic resistance in plants.

Lastly, two interesting reviews published in the Research Topic provide a valuable collection of the existing research in important aspects of defense priming. The review by Yang et al. focuses on seed priming to enhance plant disease resistance. By inducing plant immunological memory, seed priming offers a promising technique in crop protection. The authors highlight the physiological, transcriptional, metabolic, and epigenetic changes associated with defense priming and discuss the strategies and challenges in applying seed priming to enhance disease resistance in crops. The second review article focuses and summarizes the current knowledge on chemical priming of plant defense responses (Hönig et al.). Chemical priming agents have the potential to induce earlier, faster, and stronger defense responses to pathogen attacks without a minimal fitness cost. The review also highlights the involvement of key regulators, such as NONEXPRESSOR OF PR1 (NPR1) and salicylic acid signaling, in chemical priming and discusses its potential applications in enhancing plant resistance to pathogens.

Together, the published findings and revisions presented in this Research Topic contribute to the understanding of systemic resistance and defense priming against pathogens. They provide information that might be used for developing novel strategies to combat plant diseases and improve crop protection. We hope that the readers will find useful references for the systemic resistance and priming research field in this Research Topic.

## Author contributions

HJ: Funding acquisition, Writing – original draft, Writing – review & editing. NC: Funding acquisition, Writing – original draft, Writing – review & editing.

## References

[B1] Baum S. Reimer-Michalski E.-M. Bolger A. Mantai A. J. Benes V. Usadel B.. (2019). Isolation of open chromatin identifies regulators of systemic acquired resistance. Plant Physiol. 181, 817–833. doi: 10.1104/pp.19.00673 31337712 PMC6776868

[B2] Beckers G. J. M. Jaskiewicz M. Liu Y. Underwood W. R. He S. Y. Zhang S.. (2009). Mitogen-activated protein kinases 3 and 6 are required for full priming of stress responses in Arabidopsis thaliana. Plant Cell 21, 944–953. doi: 10.1105/tpc.108.062158 19318610 PMC2671697

[B3] Cecchini N. M. Jung H. W. Engle N. L. Tschaplinski T. J. Greenberg J. T. Society P.. (2015). ALD1 regulates basal immune components and early inducible defense responses in Arabidopsis. Mol. Plant Microbe Interact. 28, 455–466. doi: 10.1094/MPMI-06-14-0187-R 25372120

[B4] Conrath U. (2011). Molecular aspects of defence priming. Trends Plant Sci. 16, 524–531. doi: 10.1016/j.tplants.2011.06.004 21782492

[B5] Conrath U. Beckers G. J. M. Langenbach C. J. G. Jaskiewicz M. R. (2015). Priming for enhanced defense. Annu. Rev. Phytopathol. 53, 97–119. doi: 10.1146/annurev-phyto-080614-120132 26070330

[B6] De Kesel J. Conrath U. Flors V. Luna E. Mageroy M. H. Mauch-Mani B.. (2021). The induced resistance lexicon: do’s and don’ts. Trends Plant Sci. 26, 685–691. doi: 10.1016/j.tplants.2021.01.001 33531282

[B7] Jiang S.-C. Engle N. L. Banday Z. Z. Cecchini N. M. Jung H. W. Tschaplinski T. J. (2021). ALD1 accumulation in Arabidopsis epidermal plastids confers local and non-autonomous disease resistance. J. Exp. Bot. 72, 2710–2726. doi: 10.1093/jxb/eraa609 33463678 PMC8006555

[B8] Jung H. W. Tschaplinski T. J. Wang L. Glazebrook J. Greenberg J. T. (2009). Priming in systemic plant immunity. Science 324, 89–91. doi: 10.1126/science.1170025 19342588

[B9] Martinez-Medina A. Flors V. Heil M. Mauch-Mani B. Pieterse C. M. J. J. Pozo M. J.. (2016). Recognizing plant defense priming. Trends Plant Sci. 21, 818–822. doi: 10.1016/j.tplants.2016.07.009 27507609

[B10] Návarová H. Bernsdorff F. Döring A.-C. Zeier J. (2012). Pipecolic acid, an endogenous mediator of defense amplification and priming, is a critical regulator of inducible plant immunity. Plant Cell 24, 5123–5141. doi: 10.1105/tpc.112.103564 23221596 PMC3556979

[B11] Ryals J. Neuenschwander U. H. Willits M. G. Molina A. Steiner H. Y. Hunt M. D. (1996). Systemic acquired resistance. Plant Cell 8, 1809–1819. doi: 10.1105/tpc.8.10.1809 12239363 PMC161316

[B12] Sharrock J. Sun J. C. (2020). Innate immunological memory: from plants to animals. Curr. Opin. Immunol. 62, 69–78. doi: 10.1016/j.coi.2019.12.001 31931432 PMC7067670

[B13] Sheikh A. H. Nawaz K. Tabassum N. Almeida-trapp M. Mariappan K. G. Alhoraibi H.. (2023). Linker histone H1 modulates defense priming and immunity in plants. Nucleic Acids Res. 51, 4252–4265. doi: 10.1093/nar/gkad106 36840717 PMC10201415

[B14] Tateda C. Zhang Z. Shrestha J. Jelenska J. Chinchilla D. Greenberg J. T. (2014). Salicylic acid regulates Arabidopsis microbial pattern receptor kinase levels and signaling. Plant Cell 26, 4171–4187. doi: 10.1105/tpc.114.131938 25315322 PMC4247590

[B15] Tsuda K. Somssich I. E. (2015). Transcriptional networks in plant immunity. New Phytol. 206, 932–947. doi: 10.1111/nph.13286 25623163

[B16] Zimmerli L. Jakab G. Metraux J. P. Mauch-Mani B. (2000). Potentiation of pathogen-specific defense mechanisms in Arabidopsis by beta -aminobutyric acid. Proc. Natl. Acad. Sci. U. S. A. 97, 12920–12925. doi: 10.1073/pnas.230416897 11058166 PMC18865

